# Biographical

**Published:** 1896-05

**Authors:** 


### Biographical.

DIED, April 18th, in Paducah, Ky., Dr. J. H. Kinny. Dr. Kinny had been ill for several months, his health gradually failing. He was buried in St. Louis Cemetery, Louisville, Ky. Dr. Kinny was one of the well-known men in his profession in southwestern Kentucky, and indeed, was favorably known throughout the South. His death will be a shock to his many friends.

Dr. Kinny was born July 15th, 1842, in Brooklyn, N. Y., and received his education in that city. In 1854 he moved to Kentucky, located in Louisville, where he took a course in a dental college, after which he removed to Hardinsburg, Ky., and in 1871 removed to Paducah, Ky. He attained a good degree of professional success. The removal of such a man is a great loss to our profession. His family will have the sincere sympathy of all who knew him.

RESOLUTIONS OF RESPECT.

At a meeting of the dental profession of Paducah, held at the office of Dr. Pitcher, to take action on the death of Dr. J. H. Kinny, the following resolutions were passed:

WHEREAS, It has pleased Almighty God in His wise providence to remove from our midst our beloved practitioner, Dr. J. H. Kinny; therefore, be it

*Resolved*, That in the death of Dr. Kinny the profession at large has lost one of its most skillful and energetic members as well as a true, kind, noble and generous gentleman, one who was always ready to extend a helping hand to his fellow-dentists as well as his fellow-man, and willing with his knowledge to aid at all times the elevation of his profession.

To his beloved wife we extend our deepest heartfelt sympathy and cheer her in the belief that he has gone to his heavenly home, where he is free from pain and where there is no sorrow in that house not built with hands, eternal in the heavens.

*Resolved*, That a copy of these resolutions be sent to his wife, to each of the city papers and to different dental journals.

C. E. WHITESIDES, W. H. PITCHER,
W. L. HANSBRO, A. S. DABNEY.

PADUCAH, KY., April 20th, 1896.

